# Analytical dosimetric study of intensity-modulated radiotherapy (IMRT) and volumetric-modulated arc therapy (VMAT) for prostate cancer

**DOI:** 10.1007/s00432-023-04586-5

**Published:** 2023-01-27

**Authors:** Fady Samir, Talaat M. Meaz, Fathi AEl Hussiny, Ahmed A. Ahmed, Amr A. Mahmoud, Tamer Refaat, Ahmed Gawish, Mohamed Abouegylah

**Affiliations:** 1 Alexandria Clinical Oncology Department, Alexandria University, Alexandria, Egypt; 2 Physics Department Faculty of Science, Tanta University, Tanta, Egypt; 3 Ayadi Almostakbal Oncology Center, Alexandria, Egypt; 4 Department of Clinical Oncology, Kafr Elsheikh University, Kafr El-Sheikh, Egypt; 5 Department of Radiation Oncology, Loyola University Chicago, Chicago, USA; 6 Department of Radiation Oncology, University Hospital Magdeburg, Magdeburg, Germany

**Keywords:** Prostate cancer, IMRT, Arc, Radiotherapy planning, Organ at risk, Dosimetric evaluation

## Abstract

**Purpose:**

The study aimed to compare the dosimetric results and treatment delivery efficiency among four techniques to explore the preferred technique in prostate treatment.

**Materials and methods:**

7 IMRT, 9 IMRT, 1 ARC, and 2 ARC plans were created for 30 prostate cancer patients using the Eclipse™ treatment planning system (Varian Medical Systems). All the plans were designed to deliver 80.0 Gy in 40 fractions to the prostate planning target volume (PTV). Target coverage, organs at risk (OARs), number of monitor units, homogeneity, and conformity were compared across the four techniques to assess the quality of the plans.

**Results:**

The study revealed better Planning Target Volume (PTV) dose coverage in the VMAT-2A than in the other plans. At the same time, VMAT-2A plans were found to be significantly lower in terms of Bladder and rectum doses than other techniques. In addition, VMAT has the advantage of considerably reducing the number of monitor units and treatment time.

**Conclusion:**

For prostate cancer, VMAT may offer a favorable dose gradient profile, conformity, and MU and treatment time compared to IMRT.

## Introduction

In 2020, there will be 1.4 million new prostate cancer (PC) instances, resulting in 375,000 fatalities globally. According to Globocan 2020, it will be the fifth most common cancer among men to cause mortality worldwide in 2020. Over the past ten years, PC’s incidence and mortality have consistently grown, which is tied to aging. External beam radiation therapy is recommended for low-risk patients, and the outcomes are equivalent to surgery and brachytherapy. (D’Amico et al. 1998) It can also be used in conjunction with short-term androgen deprivation therapy (for 3 to 6 months) for intermediate-risk patients (D’Amico et al. 2004; Denham et al. 2005) and long-term androgen deprivation therapy for high-risk patients (for 2–3 years) (Bolla et al. 2002).

The advantage of radiotherapy dose escalation has been confirmed by several randomized trials, which demonstrate improved disease-free survival with high-dose radiation treatment (74–80 Gy) in comparison to standard radiation doses (68–70 Gy). (Peeters et al. 2006; Beckendorf et al. 2008) With the advancement of new radiation technologies like intensity-modulated radiotherapy (IMRT) and volumetric-modulated arc treatment (VMAT), which enable concave dose distributions around the target volumes, this dose escalation became simpler to implement. Compared to Three-Dimensional (3D) Conformal Radiotherapy, the targeted therapeutic dose is spread more accurately. Smaller doses are given to the usual structures, sparing the organs at risk, primarily the rectum and bladder (Su et al. 2005).

In IMRT, seven to nine static fields generally converge toward an iso-center at the target volume. The inverse planning system begins to function by applying various limitations, such as dosage/volume, resulting in a highly optimized dose distribution. (Chauvet et al. 2004) The mobility of the MLC leaves in the IMRT approach using static beams may happen continuously (sliding window technique) or sequentially (step and shoot technique). (Galvin et al. 1993) Karl Otto used the name VMAT, or volumetric-modulated arc treatment, to describe a novel circular irradiation approach developed from Yu’s intensity-modulated arc therapy (IMAT), which was first used in 1995. (Webb 1992) The MLC leaves’ variable speed displacement, the gantry’s variable speed rotational displacement, the dose rate’s fluctuation, and the collimator’s rotation all define the VMAT approach.

Our work aimed to determine a dosimetric evaluation method to assess the sparing of OARs in modern radiation treatments. Then, using the assessment approach, we choose a radiation strategy for prostate cancer that spares most OARs.

## Materials and methods

We included thirty prostate cancer patients in our study. Four treatment plans were carried out to achieve an optimum plan for each patient’s specific target and organs at risk. The first treatment plan was performed using the 7-field IMRT technique; the second was done using the 9-field IMRT technique; the third was done using a single arc (VMAT-1A); and the fourth was done using two arcs (VMAT-2A). These techniques were designed using Varian Eclipse treatment planning system.

This study was carried out in our center between March 2019 and January 2021. The inclusion criteria were: Age less than 80 years and histologically confirmed prostatic adenocarcinoma. Low- or intermediate-risk localized prostate cancer was according to NCCN risk classification. Low-risk patients included cT1c–T2a, N0, M0, Gleason score 6 or less, and prostate-specific antigen (PSA) concentration < 10 ng/mL. Intermediate-risk patients had at least one of the following criteria: T2b-c, Gleason score 7, and PSA 10–20 ng/mL. The exclusion criteria were: T3,4 lesions, seminal vesicle invasion, positive lymph node metastasis, Gleason score ≥ 8, risk of pelvic lymph node involvement > 15% by Roch formula, PSA > 20 ng/dl, metastatic disease, previous surgery or radiotherapy to the pelvis. All patients had baseline PSA and MRI pelvis before starting treatment.

Before simulation or delivering EBRT for prostate cancer, the patients were immobilized to maximize accuracy and minimize the movement of the target organ (i.e., the prostate) using knee support and a mattress. Patients were instructed to empty the rectum using an enema at night before the simulation. The anterior–posterior diameter of the rectum should be less than 4 cm during simulation. Also, a comfortably full bladder (the patients empty the urinary bladder, then drink 500 ml water and abstain from urination for half hour before simulation). For a setup error, the patient must be optimally positioned and immobilized to allow the target volumes to be treated with smaller margins. The patients were scanned in the CT simulator in the supine position with knee support. The slices were taken from the upper border of the L-4 vertebral body to 3 cm below the level of the lesser trochanter of the femur. The slice spacing was 2 mm over the entire treatment area. The CT data were transferred to a computerized treatment planning system where the target volumes and organs at risk were outlined according to RTOG contouring guidelines.

The planning target volumes and OARs were delineated by the radiation oncologist on the CT slices using the contouring tool of Varian Eclipse treatment planning system. Various volumes of interest were defined: GTV (prostate), CTV (prostate and proximal seminal vesicle), and PTV (CTV + 1.0 cm margin except for 7 mm posterior) were outlined as well as organs at risk (OARs), including rectum; bladder, penile bulb and the left and right head of femur were outlined. The bladder and rectum were defined by contouring the whole organ, including the contents. After simulation and contouring, the target volumes were planned on Varian eclipse software using the AAA algorithm. Four sets of plans (7F-IMRT, 9F-IMRT, VMAT-1A, and VMAT-2A) were generated and compared for this study. All four plans were done using 6MV photons.

Four treatment plans were generated for each patient by one dosimetrist to ensure plan quality and consistency across all the patients. The prescription dose for all plans was 80 Gy in 40 fractions. The 7F IMRT was carried out using the dynamic shoot technique with seven non-opposing fields at gantry angles 25, 76, 127, 181, 232, 283 and 334^°^. The 9F IMRT was carried out using a dynamic shoot technique with nine non-opposing fields at gantry angles 20, 60, 100, 140, 181, 220, 260, 300 and 340^°^. For VMAT-1A, one full arc rotation delivers radiation treatment. The gantry start angle was 180^°^, and the stop angle was 179^°^. For the two-arc technique, the first arc utilized one clockwise (CW) rotation to deliver radiation treatment. The gantry start angle was 180^°^, and the stop angle was 179^°^. The VMAT-2A employed one counterclockwise (CCW) rotation to provide radiation treatment. The gantry start angle was 180E^°^ and the stop angle was 179^°^.

**Fig. 1:**
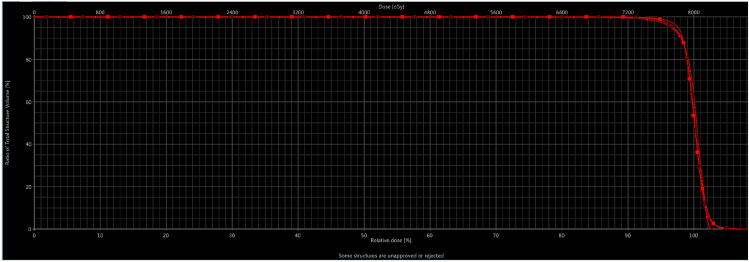
DVH of target volume coverage in one ARC, two ARC, 7F IMRT, AND 9F IMRT plans in patients with prostate cancer

Fixed optimization parameter was used in the four plans, 80 Gy for PTV. For the rectum, the dose of 60–35 Gy was specified to be 17–35% of the rectum volume, and no volume should receive more than 76 Gy. For the bladder, the dose of 45–34 Gy was specified to be 25–50% of its volume, and no volume should receive more than 76 Gy. For the penile bulb, 40 Gy was restricted to 17% of the penile bulb volume. For the left and right head of the femur, 30 Gy was specified to be 9% of the volume, and no volume should receive more than 40 Gy (Fig. 1).

**Fig. 2:**
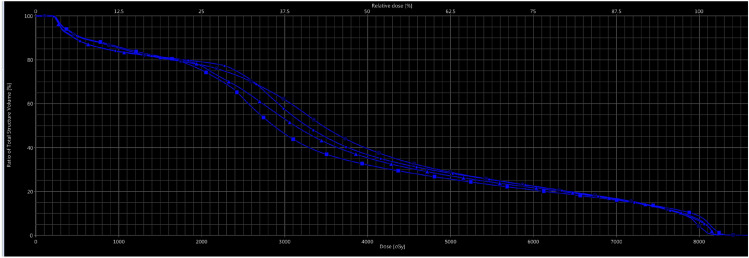
DVH of rectum dose in one ARC, two ARC, 7F IMRT, AND 9F IMRT plans in patients with prostate cancer

**Fig. 3:**
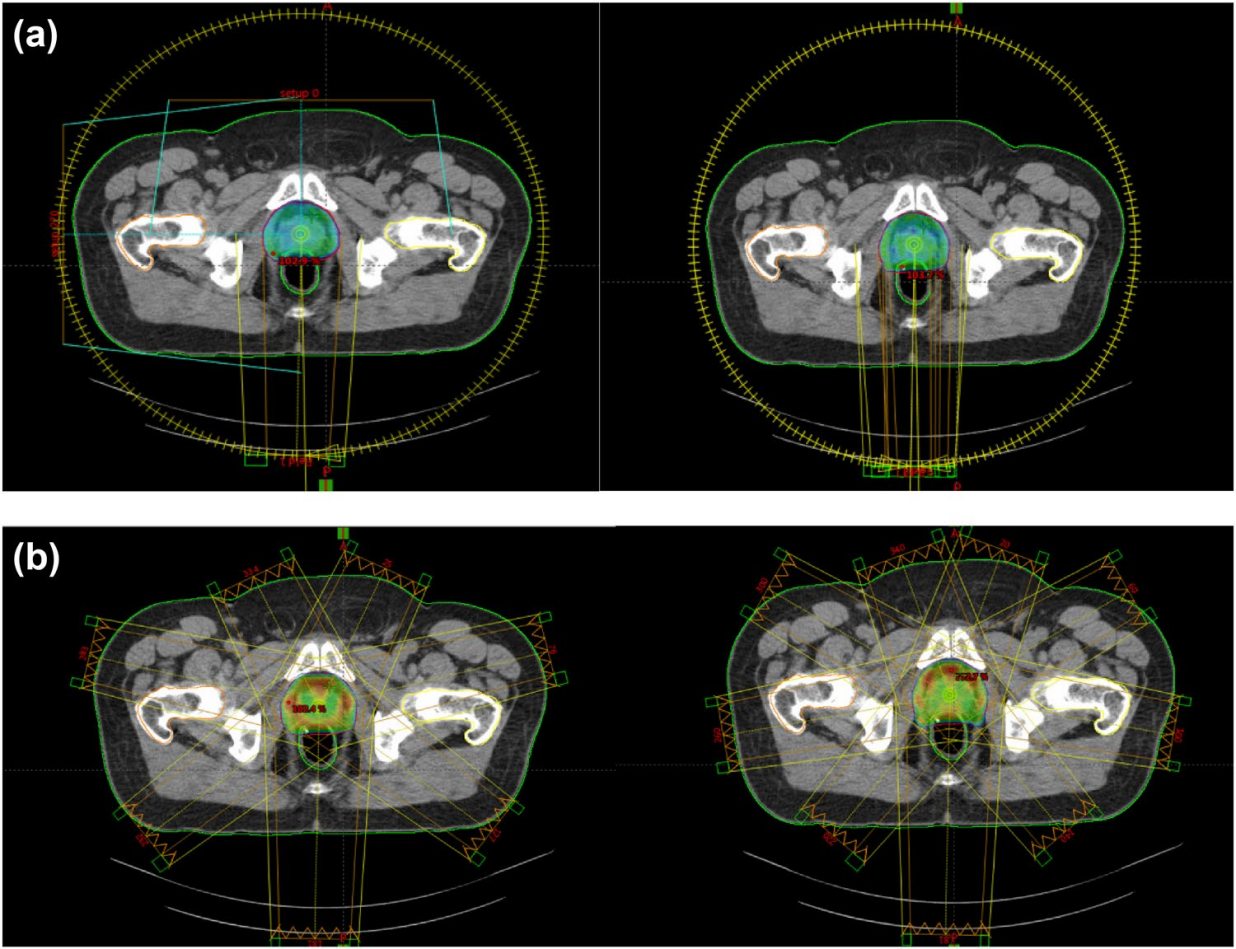
**a** Treatment plan of the same patient with one arc and two arcs. **b** Treatment plan of the same patient with 7-Feld IMRT and 9-Feld IMRT

The plans were evaluated qualitatively by comparing the dose distribution through the patient volume (cut-by-cut) and quantitatively using DVHs. The maximum dose, mean dose, and a set of values (Dx%), the percentage dose received by the *x*% volume of the target volume, and (*V*x %) the percentage volume irradiated by *x*% of the PD were obtained for the OARs. To achieve the target coverage and normal tissue sparing, the previous parameters were used (Figs. 2, 3).

Data were analyzed using SPSS version 22. Numerical data were summarized as median and range. The ANOVA test was used to determine statistical differences between volumes and doses in 7F IMRT vs. 9F IMRT vs. one ARC vs. two ARC plans. A probability (*p*-value) equal to or less than 0.05 is considered significant.

## Results

This study included thirty prostate cancer patients with a mean age of 65 years. The prostate cancer patients were at low or intermediate risk. We compared VMAT-1A, VMAT-2A, 7F-IMRT, and 9F-IMRT techniques based on two different evaluation methods named: qualitative and quantitative evaluation methods. In the first qualitative method, we used axial, sagittal, and coronal cuts and a beam eye view (BEV) from the four planning techniques for every plan in this study. In the quantitative method, we used Dose Volume Histograms (DVHs) of the four planning techniques.

The comparison between means ± SD of four groups for V95% showed that the 2-ARC technique has significantly better coverage than the other three techniques (*p* = 0.003). Likewise, the homogeneity index (HI) showed that the best technique is VMAT-2A, with a significant *P* value of 0.04. Moreover, the conformity index (CI) showed a trend toward significantly better in the 2-ARC compared to the other techniques (*p* = 0.09) Table 1.

**Table 1:** Mean ± SD of dosimetric parameters of target volume coverage in one ARC, two ARC, 7F IMRT, AND 9F IMRT plans in patients with prostate cancer

Dosimetric Parameters PTV	VMAT-1A N (30)	VMAT-2A N (30)	7F IMRT N (30)	9F IMRT N (30)	*P* value
V95%	98.94 ± 0.84	99.34 ± 0.66	98.60 ± 0.87	98.70 ± 0.90	0.003
Conformity index CI	0.99 ± 0.008	0.993330 ± 0.007	0.976161 ± 0.052	0.987002 ± 0.009	0.09
Homogeneity index HI	0.064389 ± 0.015	0.053939 ± 0.015	0.062461 ± 0.014	0.059462 ± 0.015	0.04

Regarding the rectal dose, the mean dose and the integral dose (ID) comparison showed that the VMAT-2A was insignificantly better (*P* = 0. 96 and *P* = 0.33 for the ID and mean dose, respectively) than 9F IMRT, VMAT-1A, and 7F IMRT. Also, the VMAT-2A technique significantly reduced the V30Gy, V40Gy, V50Gy, and V60Gy compared to other techniques (*P* < 0.001, 0.004, 0.003, and 0.02 respectively). Besides, the D_max_ showed that the 7F IMRT has a significantly lower dose than the other techniques (*P* < 0.001). Other dosimetric parameters are illustrated in the Table 2.

**Table 2:** Mean ± SD of dosimetric parameters of rectum in one ARC, two ARC, 7F IMRT, AND 9F IMRT plans in patients with prostate cancer

Dosimetric Parameters RECTUM	VMAT-1A N (30)	VMAT-2A N (30)	7F IMRT N (30)	9F IMRT N (30)	*P* value
Integral dose ID	2.71 ± 0.88	2.61 ± 0.87	2.78 ± 0.87	2.62 ± 0.83	0.96
MEAN	34.11 ± 5.25	32.92 ± 5.21	35.39 ± 5.51	34.03 ± 4.72	0.33
MAX	82.87 ± 1.37	83.13 ± 0.89	81.43 ± 0.56	81.44 ± 0.53	0.00
V30GY	51.9 ± 9.2	45.98 ± 8.24	56.47 ± 10.9	51.15 ± 8.4	0.00
V40GY	33.94 ± 6.0	30.04 ± 5.48	35.96 ± 7.78	33.8 ± 5.36	0.004
V50GY	24.47 ± 4.92	22.08 ± 4.99	26.78 ± 5.33	25.22 ± 4.26	0.003
V60GY	17.99 ± 4.55	16.36 ± 4.78	19.95 ± 4.99	19.14 ± 4.32	0.02
V65GY	15.28 ± 4.37	14.01 ± 4.58	16.91 ± 4.89	16.39 ± 4.28	0.07
V70GY	12.67 ± 4.15	11.77 ± 4.3	13.5 ± 4.49	13.45 ± 4.18	0.36

For the bladder dose, our results showed that the VMAT-2A technique has better lower doses to the bladder compared to the other techniques, but the difference was not statistically significant. On the other hand, the D_max_ was significantly lower in the 7F-IMRT technique (*P* = 0.001). Other dosimetric parameters for the bladder are illustrated in Table 3. The penile bulb dose showed no significant difference between the four treatment techniques with insignificant lower doses in the 7F-IMRT technique Table 4.

**Table 3:** Mean ± SD of dosimetric parameters of bladder in one ARC, two ARC, 7F IMRT, AND 9F IMRT plans in patients with prostate cancer

Dosimetric Parameters BLADDER	VMAT-1A N(30)	VMAT-2A N(30)	7F IMRT N(30)	9F IMRT N(30)	*P* value
ID	6.20 ± 2.263309	6.19 ± 2.32	6.23 ± 2.44	6.20 ± 2.43	1.00
MEAN	32.14 ± 11.12	32 ± 10.53	34.34 ± 10.43	33.37 ± 13.65	0.97
MAX	84.03 ± 1.48	83.87 ± 1.27	81.79 ± 0.31	81.82 ± 0.33	0.00
V30GY	43.12 ± 18.44	42.72 ± 16.97	44.73 ± 17.53	43.2 ± 17.32	0.98
V40GY	35.12 ± 14.67	34.11 ± 13.75	36.54 ± 14.14	36.09 ± 13.81	0.91
V50GY	28.3 ± 11.88	27.4 ± 11.15	29.95 ± 11.33	29.42 ± 11.21	0.82
V60GY	22.86 ± 9.67	22.3 ± 9.14	24.28 ± 9.05	23.96 ± 8.9	0.82
V65GY	20.52 ± 8.71	20.11 ± 8.33	21.68 ± 8.16	21.5 ± 7.95	0.86
V70GY	18.2 ± 7.83	17.97 ± 7.6	18.99 ± 7.39	18.89 ± 7.15	0.94

**Table 4:** Mean ± SD of dosimetric parameters of penile bulb in one ARC, two ARC, 7F IMRT, AND 9F IMRT plans in patients with prostate cancer

Dosimetric Parameters PB	VMAT-1A N(30)	VMAT-2A N(30)	7F IMRT N(30)	9F IMRT N(30)	*P* value
ID	0.14 ± 0.072	0.14 ± 0.07	0.13 ± 0.06	0.13 ± 0.06	0.94
MEAN	29.87 ± 10.3	30.21 ± 9.39	28.82 ± 7.92	28.87 ± 8.17	0.91
MAX	72.79 ± 13.97	72.37 ± 13.68	69.42 ± 13.12	69.11 ± 13.27	0.61
V30GY	39.5 ± 19.72	40.51 ± 16.77	39.35 ± 14.75	41.5 ± 19.05	0.96
V40GY	28.21 ± 16.98	28.31 ± 14.93	26.64 ± 13.11	26.37 ± 13.91	0.93
V50GY	19.51 ± 14.89	19.08 ± 13.99	16.87 ± 12.17	16.77 ± 12.74	0.79
V60GY	11.93 ± 12.2	11.68 ± 12.38	9.21 ± 10.06	9.29 ± 10.26	0.672
V65GY	8.7 ± 10.77	8.66 ± 11.16	6.38 ± 8.5	6.44 ± 8.65	0.66
V70GY	6.02 ± 9.05	6.06 ± 9.51	4 ± 6.55	4.09 ± 6.7	0.61

Regarding the left femur dose, the results showed that the integral dose and mean dose are significantly lower in the 7F-IMRT technique (*p* < 0.001 and *P* < 0.001, respectively), while the D_max_ was significantly better in the 9F-IMRT compared to the other techniques (*p* = 0.02). The right femur also showed that the integral dose and mean dose are markedly lower in the 7F-IMRT technique (*p* < 0.001 and 0.01, respectively). At the same time, the D_max_ was not statistically significantly different between the four techniques (*p* = 0.76). Other dosimetric parameters are illustrated in Table 5.

**Table 5:** Mean ± SD of dosimetric parameters of left femur and right femur in one ARC, two ARC, 7F IMRT, AND 9F IMRT plans in patients with prostate cancer

Dosimetric Parameters LT FEMUR	VMAT-1A N (30)	VMAT-2A N (30)	7F IMRT N (30)	9F IMRT N (30)	*P* value
ID	3.41 ± 0.62	3.22 ± 0.52	2.64 ± 0.46	2.83 ± 0.51	< 0.001
MEAN	18.23 ± 3.48	17.62 ± 3.2	14.13 ± 2.85	15.93 ± 3.53	< 0.001
MAX	40.67 ± 4.5	37.95 ± 3.28	39.8 ± 4.29	37.50 ± 5.64	0.02
V30GY	12.04 ± 9.72	7.97 ± 6.08	10.78 ± 7.12	5.30 ± 6.75	0.003
V40GY	0.37 ± 0.82	0.003 ± 0.018	0.23 ± 0.46	0.1 ± 0.24	0.02

Dosimetric Parameters RT FEMUR	VMAT-1A N (30)	VMAT-2A N (30)	7F IMRT N (30)	9F IMRT N (30)	*P* value
ID	3.13 ± 0.63	3.42 ± 0.55	2.80 ± 0.490	2.97 ± 0.53	< 0.001
MEAN	16.66 ± 4.01	18.67 ± 3.63	15.27 ± 3.65	16.26 ± 4.76	0.01
MAX	39.63 ± 3.9	39.05 ± 3.502	40.4 ± 4.45	37.36 ± 6.72	0.76
V30GY	8.54 ± 8.44	11.14 ± 9.8	12.62 ± 8.17	5.81 ± 7.92	0.02
V40GY	0.13 ± 0.28	0.09 ± 0.3	0.25 ± 0.57	0.22 ± 0.57	0.47

In the non-tumor target (NTT) dose, the integral dose (ID) and the mean dose were not statistically significantly different between the four treatment techniques. In contrast, the treatment time and the monitor units delivered are substantially lower in the VMAT-1A technique compared to the other techniques (*p* < 0.001), Table 6.

**Table 6:** Mean ± SD of dosimetric parameters of non-tumor target, monitor unit and treatment time in one ARC, two ARC, 7F IMRT, AND 9F IMRT plans in patients with prostate cancer

Dosimetric Parameters NTT	VMAT-1A N (30)	VMAT-2A N (30)	7F IMRT N (30)	9F IMRT N (30)	*P* value
ID	147.48 ± 37.8	148.49 ± 38.61	144.89 ± 36.73	143.4 ± 37.06	0.95
MEAN	4.79 ± 1.05	4.83 ± 1.07	4.72 ± 1.06	4.66 ± 1.04	0.93
Monitor unit (MU)	666.57 ± 68.22	699.40 ± 39.78	822.87 ± 114.48	895.17 ± 135.39	< 0.001
Treatment time (TT)	1.11 ± 0.11	1.16 ± 0.06	1.37 ± 0.19	1.49 ± 0.22	< 0.001

## Discussion

The current study showed that, for the treatment target volume coverage, the mean doses received by PTV of prostate cancer patients in the case of V95%, CI was significantly higher in the VMAT-2A technique compared to VMAT-1A, 9F-IMRT, and 7F-IMRT techniques. But the mean dose received by PTV for HI was insignificantly better in the VMAT-2A technique compared to VMAT-1A, 9F-IMRT, and 7F-IMRT.

The better technique should achieve better and homogeneous dose distribution to the PTV. The minimum and maximum acceptable radiation doses to the PTV should be (95–107%), achieved in the four treatment techniques. However, the inter-comparison between the four planning techniques (V95%, HI, CI, and GM) proved that the radiation doses received in VMAT-2A are the best. So, VMAT-2A could achieve better PTV coverage and dose homogeneity.

Our results agree with Abu-Hijlih et al. (2020), who reported that VMAT plans had better PTV coverage, reduced normal tissue toxicity, better CI and HI, lower MUs, and shorter treatment times than IMRT in a comparative study of IMRT and VMAT for patients treated with hypo-fractionation. Furthermore, (Ishii et al. 2013) found that in comparing the quality of the Rapid Arc technique with that of 7-f IMRT and 9-f IMRT in the treatment of high-risk prostate cancer patients, all three techniques were able to meet all the plan acceptance criteria. At the same time, the Rapid Arc resulted in slightly superior conformity and homogeneity of prostate PTV.

Regarding dosimetric parameters for organs at risk, the comparison of 7F-IMRT, 9F-IMRT, VMAT-1A, and VMAT-2A treatment techniques showed that the mean doses delivered to the rectum and bladder irradiated with the VMAT-2A technique were lower than that for 7F-IMRT, 9F-IMRT, and VMAT-1A techniques. But the mean doses delivered to penal bulb irradiated with the 7F-IMRT technique were lower than that for 9F-IMRT, VMAT-1A, and VMAT-2A techniques. Moreover, in most comparisons, diametric parameters, the mean doses delivered to the left and right femurs irradiated with the 7F IMRT technique were lower than that for 9F IMRT, VMAT-1A, and VMAT-2A techniques.

All dosimetric parameters for organs at risk were still within the dose tolerance of the prostate cancer organs at risk in four treatment techniques. But, the rectum, which is extremely close to the PTV, is the most important organ at risk of prostate cancer as well as the bladder is another important organ that should be protected in prostate cancer treatment. So, OARs are better protected in the 2ARC plan.

Our results didn’t agree with (Myerhaug et al. 2012), who performed a planning comparison in 15 patient data sets between two- or three-arc VMAT and 7-f IMRT and found that VMAT has no consistent dosimetric advantage in either the PTV or the OARs when compared with IMRT. Given the results of the present study, the quantitative dosimetric difference could be slight in the transition from multiple-field IMRT to VMAT for complicated and large PTV, including pelvic lymph nodes. The smaller sample size of only 15 patients could also affect the results. Although our results demonstrated statistically significant differences in OAR sparing among the treatment techniques, the absolute differences in dosimetric parameters were slight; therefore, whether these dosimetric benefits will reflect clinical outcomes remains to be determined. In line with the current results, (Sale and Moloney 2011) reported that the irradiated volume to the rectum at doses of 40, 50, 60, and 70 Gy was significantly reduced in VMAT, compared to IMRT. In addition, Quan et al. reported that the VMAT plan performed better plan quality on bladder sparing than the IMRT plan at doses of 30, 40, 50, 60, and 70 Gy.

Regarding dosimetric parameters for healthy tissue, the comparison of 7F-IMRT, 9F-IMRT, VMAT-1A, and VMAT-2A treatment techniques showed that 7F IMRT and 9F IMRT techniques irradiated smaller volume of normal healthy tissue in the low-dose region and delivered lower integral doses than 1ARC and 2ARC techniques. However, VMAT-1A and VMAT-2A techniques irradiated a smaller volume of normal healthy tissue in the high-dose region and delivered lower integral doses than 7F-IMRT and 9F-IMRT techniques.

The inter-comparison between the four planning techniques for MUs proved that 1ARC and 2ARC have fewer MUs than 7F IMRT and 9F IMRT techniques. So, VMAT-1A and VMAT-2A techniques shortened the treatment time delivered by 7F IMRT and 9F IMRT techniques which were more comfortable for the patient and helpful in decreasing the machine workload in a busy department. Our data were compared with those published by (Mukhtar et al. 2021), who found that VMAT was more efficient and had lesser dose delivery time and MU than IMRT.

Additionally, (Matuszak et al. 2010). Reported in Monitor Unit’s context, VMAT plans are 12.2–18.5% lower in MU compared to IMRT. The treatment efficiency was also improved in our cohort of patients when comparing IMRT versus arc therapy, with reductions in “Beam ON” time and the number of MU. The average BOT decreased by 0.38–0.33 min with the single arc and two arcs, respectively, plans compared to IMRT. The benefits of reduced “Beam ON” time include faster treatment time for the patient, which may result in greater patient comfort and an increased number of patients to be treated on a machine. Also, with the treatment given in a shorter time, the probability of intra-fractional movement decreases.

From this study, the planning target volume (PTV) can be better covered using the VMAT-2A technique. On the other hand, in comparing the doses of radiation received by the organs at risk, the organs received doses lower than the limits defined by the Radiation Therapy Oncology Group (RTOG) in all four treatment planning procedures. However, comparing the four planning procedures proved that the radiation doses received in VMAT-2A are the best. In comparing the doses of radiation received by healthy tissue, 7F IMRT and 9F IMRT plans irradiated a smaller volume of normal healthy tissue in the low-dose region and delivered lower integral doses than VMAT-1A and VMAT-2A plans. However, VMAT-1A and VMAT-2A plans irradiated a smaller volume of normal healthy tissue in the high-dose region and delivered lower integral doses than 7F IMRT and 9F IMRT plans.

## Conclusion

So, from this study, we can conclude that VMAT-2A appears to improve treatment efficiency, dosimetry, and conformity for patients with low and intermediate-risk prostate cancer compared to IMRT. This could translate into increased patient quality of life and linear accelerator productivity.

## Data Availability

The datasets are available from the first author on reasonable request.

## Abbreviations

PC: Prostate cancer
OARs: Organs at risk
PTV: Planning target volume
NTT: Non-tumor target
ID: The integral dose
CCW: Counterclockwise
IMRT: Intensity-modulated radiotherapy
ARC: Volumetric-modulated arc therapy
